# The outcomes of cardiopulmonary resuscitation and their predictors during the coronavirus 2019 pandemic in Iran

**DOI:** 10.1186/s12873-023-00860-4

**Published:** 2023-08-21

**Authors:** Afshin Goodarzi, Alireza Abdi, Hooman Ghasemi, Niloofar Darvishi, Rostam Jalali

**Affiliations:** 1 Department of Prehospital Emergency, School of paramedical, Kermanshah University of Medical Sciences, Kermanshah, Iran; 2 Department of Nursing, School of Nursing & Midwifery, Kermanshah University of Medical Sciences, Kermanshah, Iran; 3 Student Research Committee, Kermanshah University of Medical Sciences, Kermanshah, Iran

**Keywords:** Cardiopulmonary resuscitation, Coronavirus disease, Return of spontaneous circulation, CPR–discharge survival

## Abstract

**Background:**

Coronavirus disease 2019 (COVID-19) can negatively affect different healthcare-related outcomes. Nonetheless, there is limited information about its effects on different healthcare-related outcomes. This study aimed at evaluating the outcomes of cardiopulmonary resuscitation (CPR) and their predictors during the COVID-19 pandemic in Iran.

**Methods:**

This cross-sectional study was conducted on 1253 patients who had undergone CPR in the emergency wards of teaching hospitals in the west of Iran from the beginning of the first wave to the end of the third epidemic wave of COVID-19 in Iran, between February 20, 2020, and January 20, 2021. Data were collected using the National CPR Documentation Forms developed based on the Utstein Style and routinely used for all patients with cardiac arrest (CA). The SPSS (v. 20.0) program was used to analyze the data through the Chi-square, Fisher’s exact, and Mann-Whitney *U* tests and logistic regression analysis.

**Results:**

Participants’ age mean was 64.62 ± 17.54 years. Age mean among participants with COVID-19 was eight years more than other participants. Most participants were male (64.09%) and had at least one underlying disease (64.99%). The total rates of the return of spontaneous circulation (ROSC) and CPR–discharge survival were respectively 15.3% and 3.8% among all participants, 20.25% and 5.17% among participants without COVID-19, and 8.96% and 2.04% among participants with COVID-19. The significant predictors of ROSC were age, affliction by COVID-19, affliction by underlying diseases, baseline rhythm, delay in epinephrine administration, and epinephrine administration time interval, while the significant predictors of CPR–discharge survival were age and baseline rhythm.

**Conclusions:**

The total rates of ROSC and CPR–discharge survival were respectively 15.3% and 3.8% among all participants. The rates of ROSC and CPR to discharge survival among patients without COVID-19 are respectively 2.26 and 2.53 times more than the rates among patients with COVID-19.

## Introduction

Coronavirus disease 2019 (COVID-19) was first diagnosed in December 2019 and rapidly turned into a pandemic. According to the latest COVID-19 weekly epidemiological update of the World Health Organization in December 04, 2022, over 641 million confirmed cases and 6.6 million deaths have been reported globally [1]. The outbreak began in Iran after the detection of the first death associated with COVID-19, on Feb 19, 2020, in Qom, a holy city in central Iran. After a short period, COVID-19 has widely spread in all other provinces in Iran [2]. From the beginning of the epidemic, the government has emphasized social distancing rather than mass quarantine [3]. A review study conducted in January–April 2020 on the data obtained from 190 countries reported that Iran was among the top ten countries respecting COVID-19 cases [4].

Although COVID-19 primarily appears with symptoms in the upper respiratory system, involvement of the cardiovascular system, particularly among patients with the history of cardiovascular disease, is one of the most serious complications of COVID-19 and can result in acute myocardial injuries and dysfunction. Together with hypoxia caused by lung involvement, cardiovascular problems can put patients with COVID-19 at risk for cardiac arrest (CA) [5, 6]. Studies confirmed the higher risk of CA among patients with COVID-19. For example, a study showed a two times increase in the prevalence of CA during the COVID-19 pandemic [7].

Cardiopulmonary resuscitation (CPR) is the only known technique for CA management among patients with and without COVID-19 [8, 9]. However, the COVID-19 pandemic has affected CPR outcomes. A meta-analysis showed a two times increase in the rate of CA-induced in-hospital deaths among patients with COVID-19 [10]. Two studies on in-hospital and out-of-hospital CA also revealed that all patients with successful CPR eventually died before hospital discharge [11, 12].

CPR is associated with the dissemination of aerosols, particularly during chest compression, airway management, and positive pressure ventilation [9]. Therefore, it exposes CPR staff to high risk for highly contagious COVID-19 and causes concerns for them respecting the risk of affliction by COVID-19. Such concerns and the necessity to use personal protective equipment (PPE) may be associated with delays in CPR onset and hence, can affect CPR outcomes [13]. Moreover, the results of some simulation trials showed that the use of PPE during CPR for patient with COVID-19 can cause fatigue for healthcare providers, undermine their ability to perform chest compression, and negatively affect CPR quality and outcomes [14, 15]. High bed occupation rate in hospitals, recruitment of novice staff to CPR teams due to the lack of experienced CPR team members during the COVID-19 pandemic, and negative psychosocial effects of COVID-19 on healthcare providers can negatively affect CPR outcomes during the COVID-19 pandemic [16, 17].

However, information on survivability of in-hospital or out-of-hospital cardiac arrest, during the COVID-19 pandemic is lacking. Having studies that quantify CPR outcomes in these patients and identify which groups (if any) are more likely to survive to hospital discharge is critical. Some previous studies into CPR quality during the COVID-19 pandemic were conducted in non-clinical settings and using simulators, while some studies just addressed the outcomes of CA. Consequently, there is limited information about CPR outcomes during the COVID-19 pandemic. This study was conducted to narrow this gap. The aim of the study was to evaluate CPR outcomes and their predictors during the COVID-19 pandemic in Iran.

## Methods

This cross-sectional study was conducted from February 20, 2020, to January 20, 2021.

### Setting and participants

**Fig. 1:**
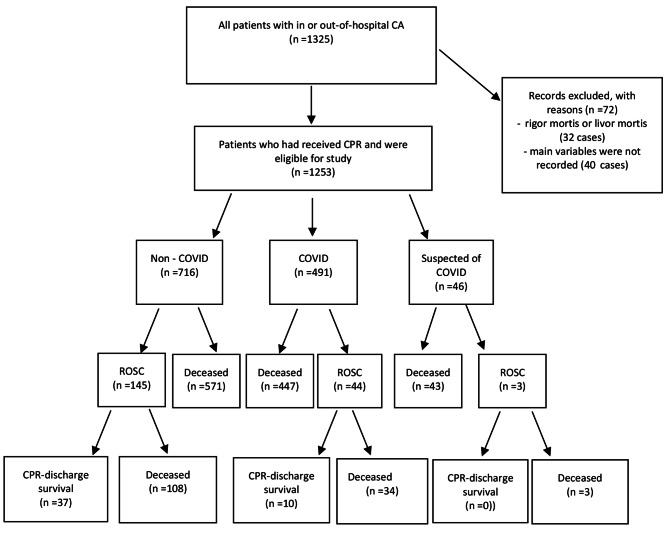
participants in the studies and their outcomes

The setting of this study was the emergency wards of teaching hospitals in the west of Iran. The required sample size was calculated based on the percentage of ROSC in a pre-epidemic study [18], 301 people, and in the present study, 1253 samples were included study for a period of 11 months. The study population consisted of patients with out-of-hospital or in-hospital CA (Fig. 1). Inclusion criteria were age over 16 years and definite diagnosis of out-of-hospital or in-hospital CA according to the Utstein Style [19, 20]. Based on the Utstein Style, in-hospital CA includes patients with a pulse rate at hospital admission who experience CA in hospital setting, while out-of-hospital CA includes patients with no pulse rate at hospital admission. This style classifies patients with CA who receive successful pre-hospital CPR as out-of-hospital CA. Patients with rigor mortis or livor mortis; also the cases for which the main variables were not recorded correctly in the CPR registration form and it was not possible to recover the information in the medical record or the hospital information system, were not included in the study.

### Data collection

According to the Utstein Style, the core criteria of successful CPR are return of spontaneous circulation (ROSC), thirty-day survival after successful CPR or live hospital discharge, and optimal neurologic status at hospital discharge or during the first thirty days after CPR [19]. In the present study, ROSC was considered as the primary CPR outcome and CPR to hospital discharge (CPR–discharge) survival was considered as the final outcome of CPR.

Study instrument was the National CPR Documentation Forms developed based on the Utstein Style and routinely used by CPR staff for all patients with CA.

The items of this form were on demographic characteristics, CPR time (08:00–14:00,14:01–20:00, 20:01–24:00, 00:01–07:59), underlying disease (Presence or absence of underlying disease and type of underlying disease), Cause of cardiac arrest (COVID-19, Poisoning, Myocardial infarction and etc.), level of consciousness at emergency ward admission (Conscious, Verbal response, Responsive to painful stimulation, Unresponsive), type of CA (in-hospital or out-of-hospital), airway management technique (Endotracheal tube, Tracheostomy, Face mask), baseline rhythm (Ventricular tachycardia, Ventricular fibrillation, Asystole, Bradycardia, Pulseless electrical activity), venous access time (Less or More than 1 min), CPR duration(Time from start to end of CPR), the number of epinephrine used, epinephrine administration time interval (< 3 min,3–5 min, > 5 min), and primary CPR outcome (ROSC or not ROSC).

The Epinephrine administration time interval was calculated by dividing the time interval between the first epinephrine administration and CPR end by the total number of epinephrine administrations [21]. This time interval was categorized as less than three minutes, 3–5 min, and more than five minutes. All patients with out-of-hospital CA (except for those who had experienced CA in the presence of prehospital emergency medical services staff) as well as patients with in-hospital CA and delayed venous access were considered as those with delayed epinephrine administration. If the CPR documentation forms were incomplete, necessary data were obtained from the patient’s medical records. Moreover, data on primary CPR outcomes and patients’ conditions at hospital discharge were collected from their electronic medical records. It should be noted that in Iranian medical centers, the quality of CPR (depth and the number of chest compressions, drugs used, application of electroshock and etc.) is routinely controlled by the resuscitation supervisor and for each CPR, a monitoring form is completed by the supervisor.

### Data analysis

Collected data were analyzed using the SPSS (v. 20.0) program. The normality of the age and the CPR duration variables was tested using the Kolmogorov-Smirnov test. Moreover, the Chi-square, Fisher’s exact tests and Mann-Whitney *U* were conducted to analyze the relationship of the primary and the final CPR outcomes with participants’ demographic characteristics, CA type, CPR time, CPR medications, use of defibrillation, venous access time, delay in epinephrine administration, epinephrine administration time interval, and CPR duration. The logistic regression analysis was also used to determine the predictors of CPR outcomes. Variables that had a relationship with CPR outcomes at a significance level of less than 0.1 were entered into the regression model together. In this model, using the ENTER method, in the Categorical Covariates Box, the sub-category that had the highest or the lowest correlation with the successful outcome of resuscitation, was selected as a reference category for other sub-categories.

### Ethical considerations

The Ethics Committee of Kermanshah University of Medical Sciences, Kermanshah, Iran approved this study (code: IR.KUMS.REC.1401.295). Necessary permissions for the study were received from the Research and Technology Administration of Kermanshah University of Medical Sciences, Kermanshah, Iran, and data collection were started after making necessary arrangements with the authorities of the study setting. Data confidentiality was ensured throughout the study. Informed consent for using patients’ medical data in research projects was routinely obtained in the study setting from patients or their first-degree family members (In patients without complete consciousness at the time of hospitalization in medical centers). This study complies with the Declaration of Helsinki and was performed according to ethics committee approval.

## Results

Among all patients who had received CPR between February 20, 2020, and January 20, 2021, 1253 patients were eligible for this study. The prevalence of COVID-19 (based on PCR or PCR and HRCT), among participants was 40.68% (n = 491) and the second leading cause of CA was myocardial infarction with a prevalence of 21.70% (n = 262). The mean participants’ age was 64.62 ± 17.54 years. The age mean among participants with COVID-19 was around eight years more than participants without COVID-19. Only 19.47% of participants with out-of-hospital CA had been transferred to hospital setting by prehospital emergency medical services and had undergone prehospital CPR. The mean CPR duration was 37.61 ± 12.02 min. Other results are reported in Tables 1 and 2.

**Table 1:** Participants’ demographic and clinical characteristics and their relationships with ROSC and CPR–discharge survival

Characteristics\CPR outcomes		Total N (%)	ROSC		P value^a^	CPR–discharge survival		P value^a^
			No	Yes		No	Yes	
Gender	Female	450 (35.91)	381 (84.67)	69 (15.33)	0.994	438 (97.33)	12 (2.67)	0.087^*^
	Male	803 (64.09)	680 (84.68)	123 (15.32)		768 (95.64)	35 (4.36)	
Age (Years)	16–24	40 (3.19)	33 (82.5)	7 (17.5)	< 0.001^*^	35 (87.5)	5 (12.5)	0.013^*^
	25–44	133 (10.61)	98 (73.68)	35 (26.32)		122 (91.73)	11 (8.27)	
	45–64	373 (29.76)	308 (82.57)	65 (17.43)		358 (95.98)	15 (4.02)	
	> 65	707 (56.42)	622 (87.98)	85 (12.02)		691 (97.74)	16 (2.26)	
Cause of cardiac arrest	COVID-19	487)40.35)	443 (90.96)	44 (9.04)	N/A^b^	477 (97.95)	10 (2.05)	0.001^*^
	COVID-19 and poisoning	4 (0.33)	4 (100)	0 (0)		4 (100)	0 (0)	
	Poisoning	59 (4.89)	39 (66.10)	20 (33.90)		49 (83.05)	10 (16.95)	
	Myocardial infarction	262 (21.70)	209 (79.77)	53 (20.23)		246 (93.89)	16 (6.11)	
	Cerebrovascular accident	62 (5.14)	54 (87.10)	8 (12.90)		62 (100)	0 (0)	
	Internal diseases	182 (15.08)	145 (79.67)	37 (20.33)		63 (100)	0 (0)	
	Sepsis	63 (5.22)	60 (95.24)	3 (4.76)		180 (98.90)	2 (1.10)	
	Surgical complications	25 (2.07)	17 (68)	8 (32)		24 (96)	1 (4)	
	Multiple trauma	42 (3.48)	29 (69.05)	13 (30.95)		35 (83.33)	7 (16.67)	
	Other	21 (1.74)	18 (85.71)	3 (14.29)		20 (95.24)	1 (4.76)	
COVID-19 Affliction	Yes	491 (40.68)	447 (91.04)	44 (8.96)	< 0.001^*^	481 (97.96)	10 (2.04)	0.708
	No	716 (59.32)	571 (79.75)	145 (20.25)		679 (94.83)	37 (5.17)	
Underlying disease	Yes	749 (65)	643 (85.85)	106 (14.15)	0.012^*^	728 (97.19)	21 (2.81)	0.073^*^
	No	403 (35)	323 (80.15)	80 (19.85)		378 (93.80)	25 (6.20)	
Type of cardiac arrest	In-hospital	986 (78.69)	830 (84.18)	156 (15.82)	0.347	948 (96.15)	38 (3.85)	0.936
	Out-of-hospital	267 (21.31)	231 (86.52)	36 (13.48)		258 (96.63)	9 (3.37)	
Baseline rhythm	Ventricular tachycardia	16 (1.3)	7 (43.75)	9 (56.25)	< 0.001^*^	12 (75)	4 (25)	0.006^*^
	Ventricular fibrillation	40 (3.2)	28 (70)	12 (30)		36 (90)	4 (10)	
	Asystole	961 (76.9)	816 (84.91)	145 (15.09)		934 (97.19)	27 (2.81)	
	Bradycardia	219 (17.50)	194 (88.58)	25 (11.42)		207 (94.52)	12 (5.48)	
	Pulseless electrical activity	13 (1)	12 (92.31)	1 (7.69)		13 (100)	0 (0)	
CPR time	08:00–14:00	307 (24.50)	255 (83.06)	52 (16.94)	0.117	296 (96.42)	11 (3.58)	0.178
	14:01–20:00	383 (30.57)	325 (84.86)	58 (15.14)		363 (94.78)	20 (5.22)	
	20:01–24:00	224 (17.88)	182 (81.25)	42 (18.75)		217 (96.88)	7 (3.12)	
	00:01–07:59	339 (27.06)	299 (88.20)	40 (11.80)		330 (97.35)	9 (2.65)	
Airway management technique	Endotracheal tube	1240 (99.1)	1050 (84.68)	190 (15.32)	0.390	1193 (96.21)	47 (3.79)	1.000
	Tracheostomy	2 (0.2)	1 (50)	1 (50)		2 (100)	0 (0)	
	Face mask	9 (0.7)	8 (88.89)	1 (11.11)		9 (100)	0 (0)	
Epinephrine delay	Yes	293 (23.52)	262 (89.42)	31 (10.58)	0.009^*^	286 (97.61)	7 (2.39)	0.788
	No	953 (76.48)	792 (83.11)	161 (16.89)		913 (95.80)	40 (4.20)	
Epinephrine administration Intervals	< 3 min	120 (9.72)	71 (59.17)	49 (40.83)	< 0.001^*^	103 (85.83)	17 (14.17)	0.110
	3–5 min	381 (30.85)	281 (73.75)	100 (26.25)		362 (95.01)	19 (4.99)	
	> 5 min	734 (59.43)	691 (94.14)	43 (5.86)		723 (98.50)	11 (1.50)	
Level of consciousness at hospital admission	Conscious	354 (29.26)	294 (83.05)	60 (16.95)	0.711	341 (96.33)	13 (3.67)	0.057^*^
	Verbal response	228 (18.84)	194 (85.9)	34 (14.91)		223 (97.81)	5 (2.19)	
	Responsive to painful stimulation	253 (20.91)	211 (83.40)	42 (16.60)		236 (93.28)	17 (6.72)	
	Unresponsive	375 (30.99)	322 (85.87)	53 (14.13)		364 (97.07)	11 (2.93)	
Type of underlying disease	1. Hypertension	91 (12.15)	77 (84.62)	14 (15.38)	N/A^b^	87 (95.60)	4 (4.40)	0.456
	2. Diabetes mellitus	75 (10.01)	66 (88)	9 (12)		74 (98.67)	1 (1.33)	
	3. Cancer	140 (18.69)	115 (82.15)	25 (17.85)		138 (98.57)	2 (1.43)	
	4. Ischemic heart disease	72 (9.61)	64 (88.89)	8 (11.11)		70 (97.22)	2 (2.78)	
	5. 1, 2, and hyperlipidemia	44 (5.87)	38 (86.36)	6 (13.64)		42 (95.45)	2 (4.55)	
	6. 1, 2, and 3	4 (0.53)	2 (50)	2 (50)		4 (100)	0 (0)	
	7. 1, 2, and 4	52 (6.94)	43 (82.69)	9 (17.31)		51 (98.08)	1 (1.92)	
	8. 1 and 4	65 (8.68)	54 (83.08)	11 (16.92)		60 (92.31)	5 (7.69)	
	9. Chronic renal failure and organ transplantation	69 (9.21)	57 (82.61)	12 (17.39)		66 (95.65)	3 (4.35)	
	10. Cerebrovascular accident	24 (3.20)	21 (87.50)	3 (12.50)		24 (100)	0 (0)	
	11. Chronic obstructive pulmonary disease	29 (3.87)	27 (93.10)	2 (6.90)		29 (100)	0 (0)	
	Other	84 (11.21)	79 (94.04)	5 (5.95)		83 (98.81)	1 (1.19)	

^a^: The results of the Chi-square or Fisher’s exact test; ^b^: Could not be computed

**Table 2:** Comparison of patients with and without covid-19 in relation to clinical - demographic characteristics

Characteristics		COVID affliction		P value^a^
		No N (%)	Yes N (%)	
Gender	Female	250(34.92)	186(37.88)	0.292
	Male	466(65.08)	305(62.12)	
Age (Years)	16–24	37(5.17)	3(0.62)	< 0.001^*^
	25–44	101(14.11)	28(5.70)	
	45–64	229(31.98)	130(26.48)	
	> 65	349(48.74)	330(67.20)	
Underlying disease	Yes	464(70.09)	272(57.63)	< 0.001^*^
	No	198(29.91)	200(42.37)	
Type of cardiac arrest	In-hospital	491(68.57)	475(96.74)	< 0.001^*^
	Out-of-hospital	225(31.43)	16(3.26)	
Baseline rhythm	Ventricular tachycardia	12(1.68)	3(0.61)	< 0.001^*^
	Ventricular fibrillation	36(5.03)	3(0.61)	
	Asystole	590(82.52)	333(67.96)	
	Bradycardia	9(1.26)	3(0.61)	
	Pulseless electrical activity	68(9.51)	148(30.20)	
CPR time	08:00–14:00	155(21.65)	131(26.68)	0.002^*^
	14:01–20:00	244(34.08)	126(25.66)	
	20:01–24:00	138(19.27)	82(16.70)	
	00:01–07:59	179(25)	152(30.96)	
Airway management technique	Endotracheal tube	714(99.72)	481(98.16)	< 0.001^*^
	Tracheostomy	2(0.28)	0(0)	
	Face mask	0(0)	9(1.84)	
Epinephrine delay	Yes	227(31.79)	39(7.99)	< 0.001^*^
	No	487(68.21)	449(92.01)	
Epinephrine administration Intervals	< 3 min	118(16.64)	2(0.41)	< 0.001^*^
	3–5 min	294(41.47)	67(13.79)	
	> 5 min	297(41.89)	417(85.80)	
Level of consciousness at hospital admission	Conscious	111(16.02)	242(50.63)	< 0.001^*^
	Verbal response	93(13.42)	133(27.82)	
	Responsive to painful stimulation	195(28.14)	52(10.88)	
	Unresponsive	294(42.42)	51(10.67)	
Type of underlying disease	1. Hypertension	58(12.50)	31(11.40)	N/A^b^
	2. Diabetes mellitus	51(10.99)	23(8.46)	
	3. Cancer	107(23.06)	31(11.40)	
	4. Ischemic heart disease	40(8.62)	29(10.66)	
	5. 1, 2, and hyperlipidemia	24(5.17)	20(7.35)	
	6. 1, 2, and 3	1(0.22)	3(1.10)	
	7. 1, 2, and 4	30(6.47)	21(7.72)	
	8. 1 and 4	45(9.70)	20(7.35)	
	9. Chronic renal failure and organ transplantation	51(10.99)	17(6.25)	
	10. Cerebrovascular accident	6(1.29)	18(6.62)	
	11. Chronic obstructive pulmonary disease	18(3.88)	10(3.68)	
	Other	33(7.11)	49(18.01)	

^a^: The results of the Chi-square or Fisher’s exact test; ^b^: Could not be computed

The rates of ROSC and CPR–discharge survival were respectively 15.3% and 3.8% among all participants, 20.25% and 5.17% among participants without COVID-19, and 8.96% and 2.04% among participants with COVID-19(Tables 1 and 2; Fig. 1). The total rate of ROSC had relationship with age (p < 0.001), affliction by COVID-19 (p < 0.001), affliction by underlying diseases (p = 0.012), baseline rhythm (p < 0.001), delay in epinephrine administration (p = 0.009), and epinephrine administration time interval (p < 0.001) at a significance level of less than 0.1. According to Mann-Whitney *U* test, there was no significant relationship between duration of resuscitation and CPR–discharge survival (p = 0.132). Moreover, the total rate of CPR–discharge survival had relationship with gender (p = 0.087), age (p = 0.013), baseline rhythm (p = 0.006), affliction by underlying diseases (p = 0.073), level of consciousness at hospital admission (p = 0.057), and the cause of CA (p = 0.001) at a significance level of less than 0.1 (Table 1). These variables were entered into the logistic regression analysis. The results of the regression analysis indicated that the significant predictors of ROSC were age, affliction by COVID-19, affliction by underlying diseases, baseline rhythm, delay in epinephrine administration, and epinephrine administration time interval, while the significant predictors of CPR–discharge survival were age and baseline rhythm (P < 0.05; Table 3).

**Table 3:** The results of the regression analysis for the predictors of ROSC and CPR–discharge survival

CPR outcomes	Independent variables	B	Std. Error	Wald	df	Sig.	Exp (B)	95% CI
ROSC	Baseline rhythm **(**Asystole**)**^**†**^	2.260	0.629	12.904	1	< 0.001^*^	9.581	2.792–32.879
	Baseline rhythm (PEA) ^**†**^	2.945	1.261	5.458	1	0.019^*^	19.010	1.607–224.880
	Baseline rhythm (Bradycardia) ^**†**^	2.318	0.666	12.104	1	0.001^*^	10.150	2.751–37.453
	Epinephrine interval **(<** 3 min) ^**‡**^	-2.652	0.298	78.964	1	< 0.001^*^	0.070	0.039-0.127
	Epinephrine interval (q3–5 min) ^‡^	-2.024	0.227	79.176	1	< 0.001^*^	0.132	0.085-0.206
	Age (25–44) ^¶^	− 0.873	0.274	3.980	1	0.047^*^	0.623	0.364-1.066
	COVID-19 Affliction (No)^¥^	− 0.986	0.240	4.721	1	0.023^*^	0.388	0.287–1.337
	Underlying disease (NO)^£^	-0.901	0.163	4.185	1	0.039^*^	0.736	0.499–1.086
	Epinephrine delay (NO)^§^	-1.339	0.244	30.054	1	< 0.001^*^	0.262	0.162–0.423
CPR–discharge survival	Age (16–24) ^¶^	-1.785	1.045	3.875	1	0.048^*^	0.168	0.022–1.302
	Baseline rhythm (Asystole) ^†^	2.783	1.117	6.213	1	0.013^*^	16.167	1.812–144.214

^**†**^. Reference category: VT; ^**‡**^. Reference category: Epinephrine interval: > 5 min; ^¶^. Reference category: >65; ^¥^. Reference category: COVID-19 Affliction; ^**£**^. Reference category: Yes; ^**§**^. Reference category: Yes

## Discussion

This study assessed CPR outcomes and their predictors in a one-year period during the COVID-19 pandemic in Iran. The total rates of ROSC and CPR–discharge survival were 15.3% and 3.8%, respectively. These rates are lower than the rates reported in studies before the COVID-19 pandemic. For example, ROSC rate in a study among patients with CA before the pandemic was 26.6% [18]. The rates of ROSC and CPR–discharge survival in other study before the pandemic were 22% and 5.2% [22], respectively. An explanation for the poorer CPR outcomes in the present study is the inclusion of patients with COVID-19 in this study. ROSC and CPR–discharge survival rates among participants without COVID-19 in our study were respectively 2.26 and 2.53 times more than the rates among patients with COVID-19. In line with our findings, previous studies reported poor CPR outcomes during the COVID-19 pandemic. For instance, a study in China reported that the primary success rate of CPR was 13.2% and thirty-day survival rate was 2.9% [23]. Two other studies also reported that none of the patients with COVID-19 who had received CPR survived to hospital discharge [11, 12]. Moreover, a meta-analysis into in-hospital CA among patients with COVID-19 showed that the cumulative CPR–discharge survival rate was 3% [10]. The fatality of CA during affliction by COVID-19, CPR team member’s concerns over affliction by COVID-19 during CPR, the necessity to wear PPE before starting CPR, and fatigue caused by using such equipment may negatively affect the quality of CPR for patients with COVID-19 [13]. Therefore, preventive measures, timely therapeutic measures, and careful patient monitoring are essential to prevent CA among patients with COVID-19 [11].

Our findings also revealed that the rates of ROSC and CPR–discharge survival among patients without COVID-19 were 20.25% and 5.17%, respectively. These CPR outcomes are poorer than those reported in studies before the COVID-19 pandemic [18, 22]. The direct and indirect physical and psychosocial effects of COVID-19 can negatively affect the chain of survival. Healthcare providers’ concerns over the possibility of affliction by COVID-19 among all newly admitted patients cause delays in their responses and services. On the other hand, high bed occupation rate during epidemic conditions is associated with changes in managerial policies in healthcare settings, increase in the need for new staff, and recruitment of novice staff to CPR teams. All these factors can have negative effects on CPR outcomes among patients without COVID-19 [16, 17, 24, 25].

Regression analysis in the present study revealed age as a significant predictor of CPR outcomes so that the lowest rates of ROSC and CPR–discharge survival were among patients over 65 years. In agreement with this finding, two studies reported significant relationship between age and CPR outcomes [26, 27]. Poorer CPR outcomes among older patients may be due to the higher prevalence of serious health problems among them and CPR team member’s poorer attitudes towards their response to CPR and post-CPR survival. Moreover, older patients may have limitations in receiving post-CPR percutaneous coronary interventions and hence, show poorer post-CPR outcomes [27]. Our findings also showed that age mean among patients with COVID-19 was eight years more than patients without COVID-19. A study reported old age as a determinant of mortality among patients with COVID-19 [28]. These findings denote the limited effectiveness of CPR among older patients with COVID-19 and highlight the importance of preventive measures such as vaccination for patients older than sixty years.

Affliction by underlying diseases was another significant predictor of ROSC in the present study and the lowest ROSC was among patients with chronic obstructive pulmonary disease. Study setting consisted of three multi-specialty referral centers with patients who mostly suffered from multiple health problems. In line with our findings, a previous study reported a significant relationship between affliction by underlying diseases and CPR outcomes [29]. Another study reported poorer CPR outcomes among patients with underlying diseases such as cancer, chronic obstructive pulmonary disease, congestive heart failure, chronic renal failure, and diabetes mellitus [30]. Contrarily, a study on 226 hospitalized patients showed no significant relationship between CPR outcomes and affliction by chronic obstructive pulmonary disease [31]. This contradiction may be due to the differences between these two studies respecting their sample size and severity of the underlying diseases.

Study findings also showed baseline rhythm as a significant predictor of ROSC and CPR–discharge survival so that the highest ROSC and CPR–discharge survival rate was among patients with shockable dysrhythmias (VT). Similarly, previous studies reported shockable dysrhythmias as a significant predictor of ROSC [26, 32, 33]. Strong evidence exists regarding higher CPR success rate among patients with shockable dysrhythmias when defibrillation is used [34]. Moreover, the rate of shockable dysrhythmias among patients without COVID-19 in the present study was 5.58 times greater than patients with COVID-19. This finding may be an explanation for poorer CPR outcomes among patients with COVID-19. Several previous studies also reported the lower rate of shockable dysrhythmias among patients with COVID-19 [11, 12, 23, 35, 36]. Hypoxia due to severe lung involvement can be a main reason for the higher prevalence of non-shockable dysrhythmias such as asystole and bradycardia among patients with COVID-19.

We also found delay in epinephrine administration as a significant predictor of ROSC. Immediate epinephrine administration is a key component of CPR guidelines [8]. In line with our findings, a study showed that the effects of epinephrine in CPR largely depend on the time of its administration and reported better CPR outcomes with earlier epinephrine administration [37]. An animal study also showed that cardiac response to epinephrine depends on its administration time [38]. The main effect of epinephrine is increase in diastolic aortic pressure subsequent to the stimulation of alpha1-adrenergic receptors in the vessels which results in increased coronary and cerebral perfusion and higher likelihood of ROSC [39].

Epinephrine administration time interval was another significant predictor of ROSC in the present study so that the rate of ROSC among patients who had received epinephrine in higher doses and shorter time intervals (i.e., less than three minutes) was higher. A meta-analysis also showed that ROSC rate among patients with standard doses of epinephrine was less than patients with high doses of epinephrine [40]. Similarly, a study reported that shorter epinephrine administration time interval was associated with better primary outcome of CPR [41]. Administration of epinephrine at high doses is associated with better coronary and cerebral perfusion and hence, is considered as a significant predictor of ROSC. Nonetheless, its effects on the long-term outcomes of CPR are still unknown because adrenaline accumulation in plasma after successful CPR can lead to tachycardia, increase myocardial need for oxygen, and thereby, cause ventricular dysrhythmias [42]. In contradiction to our findings, several studies showed that high doses of epinephrine had no significant effects or had negative effects on CPR–discharge survival [41, 43–45].

Our findings also showed that among patients with COVID-19, the highest ROSC rate was related to patients who had received epinephrine at standard doses and the lowest ROSC rate was related to patients who had received epinephrine at high doses. High doses of epinephrine can stabilize and aggravate cytokine storms caused by COVID-19. We could not find any study in this area for the purpose of comparison and hence, further studies are needed to produce more conclusive evidence.

Our study had limitations; in some cases, some information related to the resuscitation process was not available in resuscitation registration forms and patient records. It is possible for some information to be incorrectly recorded by CPR staff. Affecting the quality of resuscitation due to the staff’s concern about being infected with the Covid-19 virus due to close contact with the patient. Also, due to the nature of retrospective studies, it was not possible to control the quality of resuscitation, and the quality of resuscitation cases was confirmed only based on the reports of resuscitation supervisors and these limitations were beyond the control of the researchers.

## Conclusion

CPR outcomes among patients with COVID-19 are poorer than patients without COVID-19. The significant predictors of ROSC are age, affliction by COVID-19, affliction by underlying diseases, baseline rhythm and delay in epinephrine administration. The significant predictors of CPR–discharge survival are age and baseline rhythm. Furthermore, higher age mean, non-shockable dysrhythmias, and limited responsiveness to higher doses of epinephrine are more common among patients with COVID-19 compared with patients without COVID-19.

## Data Availability

The datasets used and/or analyzed during the current study are available from the corresponding author on reasonable request.
